# Characterization of *Candida* species isolated from clinical specimens: insights into virulence traits, antifungal resistance and molecular profiles

**DOI:** 10.1186/s12866-024-03515-x

**Published:** 2024-10-05

**Authors:** Amal F. Makled, Sahar A. M. Ali, Azza Z. Labeeb, Samar S. Salman, Doaa Z. M. Shebl, Sarah G. Hegazy, Mona S. Sabal

**Affiliations:** 1 Department of Medical Microbiology and Immunology, Faculty of Medicine, Menoufia University, Shebin al Kom, Egypt; 2 Department of Clinical Pathology, Faculty of Medicine, Menoufia University, Shebin al Kom, Egypt; 3 Department of Clinical Pharmacology, Faculty of Medicine, Menoufia University, Shebin al Kom, Egypt

**Keywords:** *Candida* species, Virulence traits, Antifungal resistance, Molecular profiles, Multiplex PCR, Clinical specimens

## Abstract

**Background:**

*Candida* species have emerged as a significant cause of opportunistic infections. Alongside the expression of various virulence factors, the rise of antifungal resistance among *Candida* species presents a considerable clinical challenge.

**Aim:**

This study aimed to identify different *Candida* species isolated from clinical specimens, evaluate their antifungal sensitivity patterns, identify key genes regulating virulence mechanisms using multiplex PCR and to assess any correlation between their virulence profiles and antifungal resistance patterns.

**Method:**

A total of 100 *Candida* spp. was isolated from 630 different clinical specimens and identified to the species level. Their antifungal susceptibility was phenotypically evaluated in accordance with CLSI guidelines using the Vitek-2 Compact System. Virulence markers, including biofilm formation capacity, protease production, melanin production, coagulase production and hemolysin production, were also phenotypically detected. The genetic determinants for biofilm formation and extracellular hydrolytic enzymes were assessed using a multiplex PCR assay.

**Results:**

The prevalence of *Candida* spp. was 15.9%, with *C. albicans* (48%) and *C. glabrata* (16%) being the most common. *C. albicans* showed the highest virulence, with strong biofilm formation, and high proteinase and melanin production. Multiplex PCR revealed Hlp in 22.0%, Hwp in 80.0%, Als in 56.0%, and Sap genes in 56.0% of isolates. Virulence genes were more common in *C. albicans* than in non-albicans *Candida* (NAC). Resistance patterns significantly correlated with virulence profiles, with notable associations between flucytosine resistance and the presence of Hlp and Hwp genes.

**Conclusion:**

The significant correlation between virulent markers such as germination, coagulase, hemolysin production and resistance patterns among different *Candida* isolates is crucial for predicting the severity and outcomes of *Candida* infections. This understanding aids in guiding tailored treatment strategies.

**Supplementary Information:**

The online version contains supplementary material available at 10.1186/s12866-024-03515-x.

## Introduction

Fungal infections affect over one billion people and cause about 1.5 million deaths annually. The lack of effective diagnostics and treatments, along with rising antifungal resistance, exacerbates the problem. At-risk patients include those with cancer, diabetes, HIV, and those using antibiotics, steroids, chemotherapy, and central venous catheters. The *Candida* genus, especially *C. albicans* and non-albicans species like *Candida auris*,* Candida glabrata*,* Candida tropicalis*, and *Candida parapsilosis*, is a major cause of severe hospital-acquired infections, and these species are high-priority targets according to the World Health Organization [1, 2].

*Candida* typically exists as a commensal organism in various body sites, including the skin, and the respiratory, gastrointestinal, and genitourinary tracts. The transition from commensalism to pathogenicity in *Candida* species is facilitated by several virulence factors. These include biofilm production, secretion of hydrolytic extracellular enzymes like proteinases, phospholipases, and hemolysin, adherence to host tissues and medical devices, and the formation of pseudohyphae [3, 4].

Clinically, different classes of antifungal agents, such as azoles, echinocandins, and polyenes, are used to treat *Candida* infections. However, the emergence of resistance to these antifungals is a growing global concern. Therefore, antifungal susceptibility testing is increasingly crucial for detecting resistance and understanding the underlying resistance mechanisms [5].

Precise identification of virulence factors in *Candida* species is essential for predicting the response to antifungal therapy and identifying emerging strains with increased resistance. The coexistence of life-threatening invasive *Candida* infections and resistance to antifungal drugs is a global concern, making it crucial to understand the relationship between virulence factors and antifungal susceptibility in *Candida.* Although there is extensive literature on the virulence and antifungal resistance of *Candida* species separately, only a few studies have explored the impact of resistance on virulence [6].

The research idea originated from the need to evaluate the prevalence of *Candida* spp. and characterize virulence markers both phenotypically and genotypically using multiplex PCR. Additionally, the phenotypic assessment of antifungal sensitivity patterns of the isolated *Candida* species was conducted. Lastly, the correlation between antifungal resistance and the virulence potential of the isolated *Candida* species was investigated.

## Patients and methods

### Study design and ethical considerations

In this cross-sectional study, one hundred *Candida* clinical isolates were obtained from 630 patients, comprising 42 males and 58 females, with a mean age of 42.60 ± 21.39 years. These patients were admitted to various departments and ICUs at Menoufia University Hospitals and the National Liver Institute between January 2022 and July 2023. The study protocol received approval from the local Ethics Committee of the Faculty of Medicine, Menoufia University (IRB number 3/21 CARD 46), and informed consent was obtained from all participating patients.

### Collection of clinical samples & identification of *Candida* Spp

*Candida* spp. was isolated from various clinical samples: blood, urine, ascitic fluid, vaginal swabs, sputum, BAL, and wound swabs, following the protocols by Britto et al. [7]. Initial identification was based on colony characteristics on Sabouraud Dextrose Agar (SDA) and microscopic examination as described by Abdelrahman and Azab [8]. Further identification and antifungal susceptibility testing were conducted using the VITEK-2 system with the Vitek ID card, YST-ID, REF 21,343 (BioMerieux, France).

### The phenotypic detection of *Candida* virulence factors was conducted as follows

#### Melanin production assay using minimal medium (MM) supplemented with L-DOPA

Melanin production was evaluated using MM containing glucose (15 mM), magnesium sulfate (10 mM), dipotassium hydrogen phosphate (29.4 mM), glycine (13 mM), and thiamine (3.0 mM), with a pH of 5.5 to 6. L-DOPA from pharmaceutical tablets was added, the pH adjusted to 5–6, sterilized, distributed onto plates. Microorganism’s colonies, previously incubated at 30 °C for 72 h on SDA, were inoculated onto the medium. Plates were incubated and observed at 24, 48, 72, and 96 h, with a final check on the 8th day, to assess melanin production, seen as black pigment, as described by de Paula Menezes et al. [9] and Almeida-Paes et al. [10].

#### Proteinase activity assessment using skimmed milk agar method

Proteinase activity was assessed using the Skimmed Milk Agar method to determine fungal protease production. Skimmed milk agar was prepared by dissolving 5 g of skimmed milk in 50 ml of distilled water and 10 g of agar in 450 ml of distilled water, both adjusted to pH 7. After sterilization and cooling to 45 °C, the solutions were combined, poured into Petri dishes, and solidified for 30 min. *Candida* species were inoculated and incubated at 37 °C for 48 h. Protein breakdown was indicated by a transparent halo around *Candida* colonies, showing zones of proteolysis, as described by Zghair [11].

#### Assessment of coagulase activity among ***Candida*** isolates

Coagulase activity in *Candida* isolates was evaluated using the test tube method. Strains were cultured overnight in Sabouraud’s dextrose broth, then 0.1 ml of each culture was added to tubes with 500 μl of rabbit, sheep, or human plasma. The tubes were incubated at 25, 37, or 45 °C and checked for clot formation at 2, 4, and 6-hour intervals, following Miruka et al. [12].

#### Evaluation oF hemolysin production among ***Candida*** isolates

Hemolysin production was evaluated by inoculating a loopful of pure *Candida* culture onto Sabouraud’s dextrose blood agar with 7% human blood and 3% glucose, followed by 48 h of incubation at 37 °C. A translucent halo around the colony indicated positive hemolysin production [13].

#### Detection of biofilm formation among ***Candida*** isolates using the tube method

For biofilm detection using the tube method, a loopful of organisms from an SDA plate was inoculated into tubes with 10 ml of Sabouraud’s dextrose broth supplemented with glucose. The tubes were incubated at 35 °C for 48 h, then washed with phosphate-buffered saline (pH 7.3) after decanting the supernatants. The dried tubes were stained with 1% crystal violet, washed with deionized water, and dried inverted. Biofilm formation was confirmed by a visible film lining the tube walls, as described by Ismail et al. [14] and Fathy et al. [15].

### Molecular detection of Hwp, Als, Sap, and Hlp virulence genes in ***Candida*** isolates was performed using multiplex PCR

DNA extraction was carried out from isolated *Candida* using The DNeasy Plant Mini Kit (Cat.No.69104) from Qiagen-Germany according to manufacturer’s instructions in a 50 μL elution volume. Two microliters of this extracted DNA were used as the template for amplifying virulence gene markers (HWP, ALS, SAP, and Hlp) by the multiplex PCR assay. The genes and primer sequences utilized are detailed in Table 1. Multiplex PCR was performed by combining 1 × PCR buffer, 0.2 mM of each of the four deoxy-nucleoside triphosphates (dNTPs), and 2 mM MgCl_2_, 2U of PlatinumTM II *Taq* Hot-Start DNA polymerase (Invitrogen, Califórnia, USA) in a 20 μL total volume. Multiplex PCR was conducted using a thermal cycler (Applied Biosystems, Singapore) following the program outlined in Table 2. The primer concentration used was adjusted according to the PCR panel used. Amplification products were visualized via agarose gel electrophoresis, and the sizes of the amplicons were determined by comparison to a 100 bp DNA ladder [16, 17].

**Table 1:** Primers sequences and amplicon sizes for multiplex PCR amplification (Invitrogen, Thermo Fisher Scientific, UK)

Genes		Primers’ sequences	Amplicon size (bp)	References
Hwp	F	5`-ATG ACT CCA GCT GGT TC- 3`	[300–500]	[16]
	R	3`-TAG ATC AAG AAT GCA GC- 5`		
Als	F	5`-GAC TAG TGA ACC AAC AAA TAC CAG A- 3`	[185–319]	[16]
	R	3`-CCA GAA GAA ACA GCA GGT GA- 5`		
Sap	F	5`-GCT CTT GCT ATT GCT TTA TTA-3`	[300–600]	[16]
	R	3`-CAT CAG GAA CCC ATA AAT CAG- 5`		
Hlp	F	5`-GTGCTGGTGACACTGCTGCT-3`	[800–1200]	[17]
	R	3`-TCCGATTCATCCACTATTTC-5`		

**Table 2:** PCR thermocycler program

Stage	Hwp	Als	Sap	Hlp
Initial denaturation	95 °C–5 min	95 °C–5 min	95 °C–5 min	95 °C–5 min
Denaturation	94 °C–30 s	94 °C–4 min	95 °C–4 min	94 °C–2 min
Annealing	50 °C–1 min	50 °C–1 min	50 °C–1 min	50 °C -1 min
Extension	72 °C–1 min	72 °C–1 min	72 °C–1 min	72 °C–1 min
Cycle number	30 cycles	30 cycles	30 cycles	30 cycles
Final extension	72 °C–5 min	72 °C–5 min	72 °C–5 min	72 °C–5 min

### Statistical analysis

The data were analyzed using SPSS software version 20. Results were presented as percentages. Chi-square tests were conducted, with significance set at a *p*-value < 0.05.

## Results

In this study, positive fungal growth was observed in 17.6% of specimens. *Candida* species were the most common (15.9%; 100/630), followed by *Aspergillus* spp. (1%; 6/630) and *Cryptococcus* spp. (0.8%; 5/630). *Candida* spp. were predominantly isolated from sputum (50%), vaginal swabs (34.3%), urine (20%), and BAL specimens (20%), with the lowest occurrence in ascitic fluid (8%), as shown in Table 3.

**Table 3:** Distribution ofCandidaspp. and other fungi isolated from 630 clinical specimens using morphological identification on SDA

Source of specimens	No. of specimens	Candida isolates		Other fungi				No growth on SDA	
				Aspergillus		Cryptococcus			
		No.	%	No.	%	No.	%	No.	%
Blood culture	400	54	13.5%	4	1%	2	0.5%	340	85%
Urine	120	24	20%	0	0%	0	0%	96	80%
Sputum	4	2	50%	0	0%	0	0%	2	50%
Wound swabs	11	2	18.2%	1	9.1%	0	0%	8	72.7%
BAL	10	2	20%	1	10%	0	0%	7	70%
Vaginal Swab	35	12	34.3%	0	0%	0	0%	23	65.7%
Ascitic Fluid	50	4	8%	0	0%	3	6%	43	86%
Total No. of specimens	630	100	15.9%	6	1%	5	0.8%	519	82.4%

*C. albicans* was predominant in candidemia cases (58.3%), while non-albicans *Candida* (NAC) species were more prevalent in candiduria cases (77.8%). Mixed cases (candidemia and candiduria) had the highest mortality rate (66.7%). A significant difference in *Candida* species distribution was observed among different infection types (*P*-value of 0.009) as shown in Table 4.

**Table 4:** Distribution of *Candida* isolates and associated infections with outcome analysis

Candida Isolates (VITEK ID)	Candida in blood Candidemia (*n* = 48)		Candida in urine Candiduria (*n* = 18)		Mixed (*n* = 6)		Candida in other organs (*n* = 28)		Total (*n* = 100)		χ2	*p*-value
	No	%	No	%	No	%	No	%	No	%		
*C.albicans*	28	58.3	4	22.2	2	33.3	14	50.0	48	48.0	45.79	0.001*
*NAC	20	41.7	14	77.8	4	66.7	14	50.0	52	52.0		
• *C.glabrata*	0	0.0	4	22.2	2	33.3	10	35.7	16	16.0		
• *C tropicalis*	6	12.5	2	11.1	2	33.3	4	14.3	14	14.0		
• *C parapsilosis*	8	16.7	2	11.1	0	0.0	0	0.0	10	10.0		
• *C.dubliniensis*	2	4.2	2	11.1	0	0.0	0	0.0	4	4.0		
• *C.guilliromondii*	2	4.2	2	11.1	0	0.0	0	0.0	4	4.0		
• *C lusitaniae*	0	0.0	2	11.1	0	0.0	0	0.0	2	2.0		
• *C.auris*	2	4.2	0	0.0	0	0.0	0	0.0	2	2.0		
Outcome							26	92.9	78	78.0	11.6	0.009*
• Alive	35	72.9	15	83.3	2	33.3	2	7.1	22	22.0		
• Died	13	27.1	3	16.7	4	66.7						

*C. albicans* was highly susceptible to all antifungals, particularly Voriconazole and Amphotericin B, with 100% susceptibility. In contrast, NAC species exhibited lower susceptibility rates across all antifungals and significant resistance to Fluconazole, Flucytosine and Caspofungin. The statistically significant *p*-values for all drugs indicate that these differences in drug susceptibility between *C. albicans* and NAC are highly significant and not due to random variation. as detailed in Table 5.

**Table 5:** Correlation between *Candida* species and antifungal drug resistance in studied patients

Antifungal drugs		Candida spp.						
		*C.albicans (n = 48)*			NAC *(n = 52)*			
		S	**R**	I	S	**R**	I	*p*-value
Azoles	Voriconazole	48 (100.0)	0 (0.0)	0 (0.0)	50 (96.2)	2 (3.8)	0 (0.0)	< 0.001*
	Fluconazole	46 (95.8)	0 (0.0)	2 (4.2)	29 (55.8)	6 (11.5)	1 (1.9)	< 0.001*
Pyrimidine analogues	Flucytosine	47 (97.9)	0 (0.0)	1 (2.1)	43 (82.7)	8 (15.4)	1 (1.9)	< 0.001*
Polyenes	Amphotericin B	48 (100.0)	0 (0.0)	0 (0.0)	50 (96.2)	2 (3.8)	0 (0.0)	< 0.001*
Echinocandins	Micafungin	44 (91.7)	4 (8.3)	0 (0.0)	46 (88.5)	4 (7.7)	2 (3.8)	< 0.001*
	Caspofungin	44 (91.7)	4 (8.3)	0 (0.0)	38 (73.1)	9 (17.3)	5 (9.6)	< 0.001*

*: Statistically significant

*C. albicans* displayed heightened levels of virulence factors, with biofilm formation being the most prevalent (37.5%). Proteinase production was notable in *C. albicans* (46.2%), as was melanin production (64.7%). Hemolytic activity was observed in half of *C. albicans* strains (50.0%). Coagulase production was prevalent in approximately 52.4% of *C. albicans* strains as indicated in Table 6.

**Table 6:** Prevalence of various virulence traits among studied *Candida* species

Candida spp.	No. of positive strains/Total no. of strains													
	Biofilm		Germination		Proteinase		Melanin		Hemolytic activity		Coagulase		Total	
	No	%	No	%	No	%	No	%	No	%	No	%	No	%
*C.albicans*	12	37.5	48	85.7	24	46.2	22	64.7	22	50.0	22	52.4	150	57.7
NAC														
• *C.glabrata*	6	18.8	0	0.0	8	15.4	8	23.5	12	27.3	6	14.3	40	15.4
• *C tropicalis*	10	31.3	4	7.1	12	23.1	2	5.9	10	22.7	10	23.8	48	18.5
• *C parapsilosis*	2	6.2	0	0.0	4	7.7	0	0.0	0	0.0	2	4.8	8	3.0
• *C.dubliniensis*	0	0.0	4	7.1	0	0.0	0	0.0	0	0.0	0	0.0	4	1.5
• *C.guilliromondii*	0	0.0	0	0.0	0	0.0	0	0.0	0	0.0	2	4.8	2	0.8
• *C lusitaniae*	0	0.0	0	0.0	2	3.8	0	0.0	0	0.0	0	0.0	2	0.8
• *C.auris*	2	6.2	0	0.0	2	3.8	2	5.9	0	0.0	0	0.0	6	2.3
Total	**32**	**100.0**	**56**	**100.0**	**52**	**100.0**	**34**	**100.0**	**44**	**100.0**	**42**	**100.0**	**260**	**100.0**

A correlation between resistance patterns and virulence profiles among *Candida* isolates, highlighting statistically significant differences in virulence markers such as germination, hemolytic activity, and coagulase production concerning resistance to antifungal drugs across different *Candida* isolates. as shown in Table 7.

**Table 7:** Correlation between antifungal resistance and virulence traits in *Candida* species

Antifungal Resistance		Virulence traits											
		Biofilm		Germination		Proteinase		Melanin		Hemolytic activity		Coagulase	
		+ve	-ve	+ve	-ve	+ve	-ve	+ve	-ve	+ve	-ve	+ve	-ve
		n (%)	n (%)	n (%)	n (%)	n (%)	n (%)	n (%)	n (%)	n (%)	n (%)	n (%)	N (%)
Azoles	Fluconazole (n = 6)	4 (66.7)	2 (33.3)	0 (0.0)	6 (100)	6 (100)	0 (0.0)	2 (33.3)	4 (66.7)	0 (0.0)	6 (100)	2 (33.3)	4 (66.7)
	Voriconazole (n = 2)	2 (100)	0 (0.0)	0 (0.0)	2 (100)	2 (100)	0 (0.0)	2 (100)	0 (0.0)	0 (0.0)	2 (100)	0 (0.0)	2 (100)
Pyrimidine analogues	Flucytosine (n = 8)	6 (75.0)	2 (25.0)	4 (50.0)	4 (50.0)	8 (100)	0 (0.0)	2 (25.0)	6 (75.0)	8 (100)	0 (0.0)	4 (50.0)	4 (50.0)
Echinocandins	Caspofungin (n = 13)	10 (76.9)	3 (23.1)	9 (69.2)	4 (30.8)	9 (69.2)	4 (30.8)	7 (53.8)	6 (46.2)	11 (84.6)	2 (15.4)	10 (76.9)	3 (23.1)
	Micafungin (n = 8)	6 (75.0)	2 (25.0)	7 (87.5)	1 (12.5)	6 (75.0)	2 (25.0)	4 (50.0)	4 (50.0)	5 (62.5)	3 (37.5)	7 (87.5)	1 (12.5)
Polyenes	Amphotericin B (n = 2)	2 (100)	0 (0.0)	0 (0.0)	2 (100)	2 (100)	0 (0.0)	2 (100)	0 (0.0)	0 (0.0)	2 (100)	0 (0.0)	2 (100)
Total		**30** **(76.9)**	**9** **(23.1)**	**20** **(51.3)**	**19** **(48.7)**	**33** **(84.6)**	**6** **(15.4)**	**19** **(48.7)**	**20** **(51.3)**	**24** **(61.6)**	**15** **(38.4)**	**23** **(59.0)**	**16** **(41.0)**
χ2		**1.59**		**16.41**		**6.20**		**6.72**		**23.93**		**12.07**	
*p*-value		**0.903**		0.006*		**0.287**		**0.242**		< 0.001*		0.033*	

*: Statistically significant, χ2: Chi-squared test

**Fig. 1:**
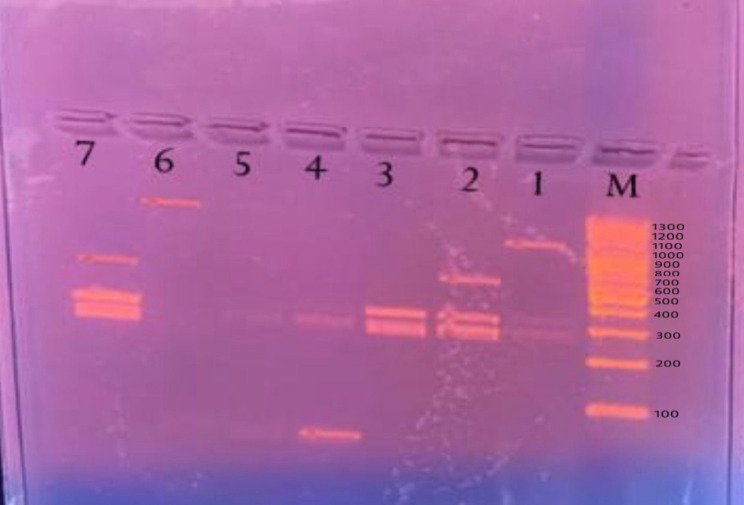
Multiplex PCR amplified products of *Candida* spp. specific virulence genes • LaneM: Molecular weight marker [100–1300 bp] • Lanes 1and 6: Hlp gene (1191 bp) among *Candida glabrata* isolate • Lanes 2 and 7: Als gene (271 bp), Hwp gene (314 bp) and Sap genes (605 bp) among *Candida glabrata* isolate • Lane 3: Als gene (271 bp) and Hwp gene (314 bp) among *Candida albicans* isolate

Ultraviolet transillumination revealed DNA bands for the Als, Sap, Hwp, and Hlp genes among *Candida* isolates. Hwp, Als, and Sap genes were detected across various *Candida* isolates, while the Hlp gene was exclusively detected among *Candida glabrata* and *Candida tropicalis* isolates as demonstrated in Fig. 1.

Additionally, 80% of *Candida* isolates were positive for Hwp genotypes, with varying representation among different *Candida* species. Similarly, 56% of *Candida* isolates were positive for both Als and Sap genotypes, with varying distributions among different *Candida* species. Statistically significant differences were observed in both Hlp and Hwp positive genotypes among *Candida* species (*P* < 0.001). Moreover, Sap positive genotypes also exhibited a statistically significant difference among *Candida* species (*P* = 0.026), as detailed in Table 8.

**Table 8:** Distribution of virulence genes among *Candida* species in studied patients

Candida Spp.	Virulence genes							
	Hlp		Hwp		Als		Sap	
	+ve (*n* = 22) No (%)	-ve (*n* = 78) No (%)	+ve (*n* = 80) No (%)	-ve (*n* = 20) No (%)	+ve (*n* = 56) No (%)	-ve (*n* = 44) No (%)	+ve (*n* = 56) No (%)	-ve (*n* = 44) No (%)
C.albicans (n = 48)	0 (0.0)	48 (100.0)	44 (91.7)	4 (8.3)	28 (58.3)	20 (41.7)	26 (54.2)	22 (45.8)
C.glabrata (n = 16)	12 (75.0)	4 (25.0)	8 (50.0)	8 (50.0)	6 (37.5)	10 (62.5)	8 (50.0)	8 (50.0)
C tropicalis (n = 14)	10 (71.4)	4 (28.6)	12 (85.7)	2 (14.3)	10 (71.4)	4 (28.6)	12 (85.7)	2 (14.3)
C parapsilosis (n = 10)	0 (0.0)	10 (100.0)	8 (80.0)	2 (20.0)	4 (40.0)	6 (60.0)	4 (40.0)	6 (60.0)
C.dubliniensis (n = 4)	0 (0.0)	4 (100.0)	2 (50.0)	2 (50.0)	2 (50.0)	2 (50.0)	2 (50.0)	2 (50.0)
C.guilliromondii (n = 4)	0 (0.0)	4 (100.0)	2 (50.0)	2 (50.0)	2 (50.0)	2 (50.0)	0 (0.0)	4 (100.0)
C lusitaniae (n = 2)	0 (0.0)	2 (100.0)	2 (100.0)	0 (0.0)	2 (100.0)	0 (0.0)	2 (100.0)	0 (0.0)
C.auris (n = 2)	0 (0.0)	2 (100.0)	2 (100.0)	0 (0.0)	2 (100.0)	0 (0.0)	2 (100.0)	0 (0.0)
Total	**22** **(22.0)**	**78** **(78.0)**	**80** **(80.0)**	**20** **(20.0)**	**56** **(56.0)**	**44** **(44.0)**	**56** **(56.0)**	**44** **(44.0)**
Test of sig.	**χ2 = 60.196**		**χ2 = 17.200**		**χ2 = 7.98**		**χ2 = 13.833**	
p-value	< 0.001*		< 0.001*		0.334		0.026*	

*: Statistically significant, χ2: Chi-squared test

Biofilm-positive *Candida* isolates exhibited prevalence rates of Hwp, Sap, Als, and Hlp genotypes at 100%, 93.8%, 87.5%, and 43.8%, respectively. Similar patterns were observed across germinating, proteinase-producing, hemolytically active, and coagulase-producing isolates. Germinating isolates showed these genotypes in 89.3%, 57.1%, 60.7% and 7.1% of cases, respectively. Proteinase-producing isolates exhibited prevalence ranging from 88.5 to 100% for Sap, Hwp, Als, and Hlp genotypes. Hemolytically active isolates displayed positivity for these genotypes in 81.8–90.9% of cases. Coagulase-producing isolates exhibited prevalence ranging from 28.6 to 85.7%, Table 9.

**Table 9:** Association between virulence traits and virulence genes in studied patients

Virulence traits	Virulence genes							
	Hlp		Hwp		Als		Sap	
	+ve (*n* = 22) *n* (%)	-ve (*n* = 78) *n* (%)	+ve (*n* = 80) *n* (%)	-ve (*n* = 20) *n* (%)	+ve (*n* = 54) *n* (%)	-ve (*n* = 46) *n* (%)	+ve (*n* = 56) *n* (%)	-ve (*n* = 44) *n* (%)
Biofilm (n = 32)	14 (43.8)	18 (56.2)	32 (100.0)	0 (0.0)	28 (87.5)	4 (12.5)	30 (93.8)	2 (6.2)
Germination (n = 56)	4 (7.1)	52 (92.9)	50 (89.3)	6 (10.7)	34 (60.7)	22 (29.3)	32 (57.1)	24 (42.9)
Proteinase (n = 52)	18 (34.6)	34 (65.4)	50 (96.2)	2 (3.8)	46 (88.5)	6 (11.5)	52 (100.0)	0 (0.0)
Melanin (n = 34)	10 (29.4)	24 (70.6)	30 (88.2)	4 (11.8)	28 (82.4)	6 (17.6)	32 (94.1)	2 (5.9)
Hemolytic activity (n = 44)	22 (50.0)	22 (50.0)	38 (86.4)	6 (13.6)	36 (81.8)	8 (18.2)	40 (90.9)	4 (9.1)
Coagulase (n = 42)	12 (28.6)	30 (71.4)	34 (81.0)	8 (19.0)	34 (81.0)	8 (19.0)	36 (85.7)	6 (14.3)
Test of sig.	**χ2 = 25.33**		**χ2 = 10.36**		**χ2 = 16.15**		**χ2 = 49.64**	
*p*-value	< 0.001*		0.065		0.006*		< 0.001*	

*: Statistically significant, χ2: Chi-squared test

Significant differences were observed in Hlp, Als, and Sap genotype prevalence among virulent traits (*p* < 0.001, *p* = 0.006, and *p* < 0.001, respectively). Hlp-positive genotypes were prevalent among various antifungal-resistant isolates, with Hwp-positive genotypes being particularly common. Additionally, Als and Sap genotypes showed similar patterns among resistant isolates, as indicated in Table 10.

**Table 10:** Relation between antifungal resistance and virulence genes in the studied patients

Antifungal Resistance		Virulence genes									
		Hlp positive		Hwp positive		Als positive		Sap positive		χ2	*p*-value
		(n = 22)		(n = 80)		(n = 54)		(n = 56)			
		No	%	No	%	No	%	No	%		
Azoles	Fluconazole	0	0	6	100	4	66.7	6	100	12.49	0.642
	(n = 6)										
	Voriconazole	0	0	2	100	0	0	2	100		
	(n = 2)										
Pyrimidine analogues	Flucytosine	8	100	8	100	6	75	8	100		
	(n = 8)										
Echocandins	Caspofungin	11	84.6	12	92.3	10	76.9	10	76.9		
	(n = 13)										
	Micafungin	6	75	7	87.5	7	87.5	6	75		
	(n = 8)										
Polyenes	Amphotericin B	0	0	2	100	0	0	2	100		
	(n = 2)										

χ2: Chi-squared test

## Discussion

*Candida* species are opportunistic fungi causing infections that range from superficial mucocutaneous diseases to life-threatening invasive systemic infections. While *Candida albicans* is the most common cause of these infections, other species like *Candida glabrata* and *Candida tropicalis* have become more prevalent in recent decades [18].

Our study found 100 *Candida* spp. isolates from 630 samples (15.9%), with *C. albicans* accounting for 48% and non-albicans *Candida* species (NAC) making up the remaining 52%. This aligned with Raja’s findings [19], where NAC was at 55% and *C. albicans* at 45%. Similar trends were reported by Ortiz et al. [20] and Malinovská et al. [21], with *C. albicans* being the predominant species at 52.4% and 50%, respectively. However, Boonslip et al. [22] and Nandini et al. [23] identified *C. tropicalis* as the most common, at 33.33% and 41% respectively.

In our study, a mortality rate of 66.7% was observed in cases of mixed candidemia and candiduria, highlighting the seriousness of polymicrobial *Candida* infections. This underscores the need for tailored therapeutic strategies to manage fungal spread and enhance patient outcomes. Comprehensive surveillance and characterization of *Candida* infections, including species identification and susceptibility testing, are essential for guiding effective antifungal treatment and reducing the risk of treatment failure or adverse effects [24].

Approximately 8.3% of *C. albicans* showed resistance to both caspofungin and micafungin, while non-albicans *Candida* species (NAC) displayed resistance rates of 26.9% to caspofungin, 17.3% to flucytosine, and 13.5% to fluconazole. Voriconazole and amphotericin B showed 100% sensitivity among *Candida* isolates, except for two *C. auris* isolates. Siddiqui et al. [1] reported similar findings with 89.1% sensitivity to voriconazole and 89% to amphotericin B among *Candida* isolates. Seyoum et al. [25] found 3.8% resistance in *C. albicans* to caspofungin and micafungin, with no resistance observed in NAC species. Chen et al. [26]. highlighted that despite 68% of *Candida*-infected patients receiving appropriate antifungal therapy, the mortality rate remained high at 56%, suggesting that virulence factor expression influences patient outcomes. Accurate detection of these factors in *Candida* species is crucial for predicting antifungal response and preventing resistant strains [26]. Virulence factors, including adhesion, colonization, and biofilm formation on medical devices, impede antifungal penetration and evade host defenses [27]. Additionally, proteases, hemolysins, and coagulases aid tissue invasion [28]. Hemolysins release iron, supporting *Candida* survival and spread [4], while coagulases activate clotting by binding to fibrinogen [13]. Furthermore, melanin production helps *Candida* evade the immune system, reducing phagocyte effectiveness and altering responses to antifungals [29].

Our study found that 32% of *Candida* isolates exhibited biofilm production, consistent with the findings of Mohammadi et al. [30] and Pramodhini et al. [31]. However, Sheena and Singh [32] reported a higher rate, with 77.1% of isolates producing biofilms. Among our biofilm producers, *C. albicans* accounted for 37.5%, followed by *C. tropicalis* (31.3%) and *C. glabrata* (18.8%). Ismail et al. [14] and Saiprom et al. [27] observed *C. tropicalis* as the most frequent biofilm producer, followed by *C. glabrata* and *C. albicans*. Conversely, David et al. [33] reported *C. glabrata* as the most frequent, followed by *C. albicans* and *C. tropicalis.*

In our research, *C. albicans* emerged as the primary isolate for germination (85.7%), proteinase (46.2%), melanin production (64.7%), hemolytic activity (50%), and coagulase production (52.4%). Similarly, Hadid and Abed [34] found *C. albicans* to be the predominant producer of proteinase, coagulase, and hemolysin. David et al. [33] also reported *C. albicans* as the primary source of hemolytic activity and melanin production. Factors influencing melanin production include temperature variations, tyrosinase enzyme production, different *Candida* clades and strains, and the efficacy of culture media [35].

Our findings, supported by Talapko et al. [36] and Tulasidas et al. [37], highlighted a correlation between *Candida* isolation sites and virulence factor expression. *Candida* spp. isolated from blood exhibited heightened virulence, expressing various virulence markers, likely due to their ability to adhere to catheters and produce extracellular enzymes for tissue invasion. However, Chen et al. [26] and Jabeen et al. [38] observed no significant difference in virulence patterns across different clinical specimen sources.

In our study, we found notable differences in virulence markers such as germination, hemolytic activity, and coagulase production related to antifungal resistance. This observation aligned with Miruka et al. [12], who reported that a most of antifungal-resistant *Candida* isolates produced protease and coagulase, and all exhibited biofilm production with hemolytic activity. This correlation suggests that resistant strains often have thicker cell walls and share genetic pathways with virulence. Factors such as germination and enzyme production, including coagulase and hemolysins, can influence resistance. *Candida* species carry essential virulence genes like Hwp, Als, and SAP genes crucial for adhesion, biofilm formation, and tissue invasion. Hemolysin production, regulated by the HLP gene, varies among *Candida* strains, affecting their pathogenicity. Detecting these genes in clinical samples is vital for understanding *Candida* colonization and disease progression [12, 38–40].

In our PCR analysis, 56% of *Candida* isolates tested positive for ALS genotypes, with *C. tropicalis* and *C. albicans* showing the highest occurrences. However, gene prevalence varied across studies, influenced by sample types and diagnostic methods, as highlighted by Ardehali et al. [41] and Ahmed et al. [40]. Similarly, SAP and Hwp genotypes were detected in 56% and 80% of isolates, respectively, with varying distributions among species compared to other research findings, as reported by Ali et al. [42] and Fathy et al. [15]. For the HLP gene associated with hemolytic activity, 22% of our isolates tested positive, with significant proportions found in *C. glabrata* and *C. tropicalis*. Variations in gene prevalence can be attributed to factors such as sample size and origin, as observed by Shrief et al. [43] and Dawoud et al. [44].

This study demonstrated genotype distribution among *Candida* isolates and its correlation with biofilm formation and virulent traits. High prevalence of Hwp, Sap, Als, and Hlp genotypes in biofilm-positive *Candida* suggests their role in biofilm formation, linked to increased antifungal resistance and persistent infections. Understanding genotypic factors enhances strategies against biofilm-related infections [30], emphasizing species-specific considerations in *Candida* pathogenicity. Statistically significant differences in genotype prevalence necessitate tailored approaches in managing *Candida* infections [45].

The association between genotype prevalence and virulent traits provides insights into *Candida*’s genetic determinants of virulence. Certain genotypes’ high prevalence across virulent traits suggests their potential role in enhancing *Candida* pathogenicity, aiding in identifying molecular targets for novel antifungal strategies [46]. Genotype diversity influences antifungal resistance, with specific genotypes potentially contributing to resistance mechanisms. Understanding this genetic basis is crucial for developing effective therapeutic strategies against antifungal resistance. Notably, fluconazole and itraconazole-resistant isolates often exhibit simultaneous presence of ALS and HWP genes, as reported by Mohammadi et al. [30], consistent with prior research by Shrief et al. [43]. Similarly, Schikora-Tamarit and Gabaldon [45] link HWP and ALS genes to antifungal resistance, associating them with adhesion and biofilm formation in *Candida* species.

## Conclusion

*Candida albicans* was identified as the predominant isolate with a higher susceptibility to antifungal agents compared to non-albicans *Candida* (NAC) species, which displayed significant resistance, particularly to azoles and echinocandins. *Candida albicans* also exhibited a diverse array of virulence traits, such as biofilm formation and melanin production, which were less common in NAC species. The study found notable correlations between antifungal resistance and the presence of specific virulence traits and genes, such as Hwp and Sap, suggesting that virulence characteristics and resistance profiles are interrelated. This highlights the need for targeted antifungal therapy and further research into the mechanisms linking virulence factors with drug resistance to improve the management of *Candida* infections.

## Electronic supplementary material

Below is the link to the electronic supplementary material.

Supplementary Material 1

Supplementary Material 2

Supplementary Material 3

Supplementary Material 4

Supplementary Material 5

## Data Availability

All data used to support the findings of this study are available from the corresponding author on request.

## Abbreviations

Als: Agglutinin like sequence
CLSI: Clinical and Laboratory Standards Institute
Hlp: Hemolysin like protein
Hwp: Hyphal wall protein
Sap: Secreted aspartyl proteases
