# Supplementary material for: Characterization of Candida species isolated from clinical specimens: insights into virulence traits, antifungal resistance and molecular profiles
Source: BMC Microbiol. 2024 Oct 5;24:388. doi: 10.1186/s12866-024-03515-x (PMC11453005; doi:10.1186/s12866-024-03515-x)
Supplement: Supplementary file 1 — Supplementary Material 1 [file 12866_2024_3515_MOESM1_ESM.docx]

**supplementary figures and tables**

| 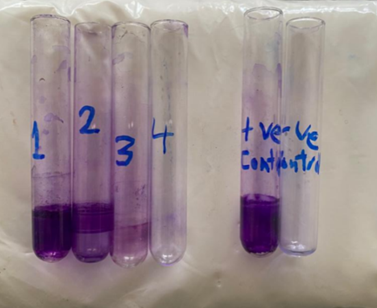  **Rt.**  **Lt.** | 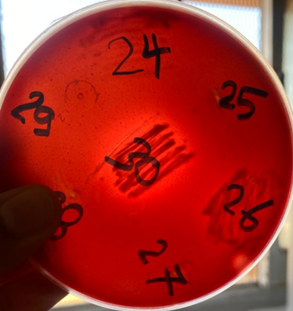 |
| --- | --- |
| 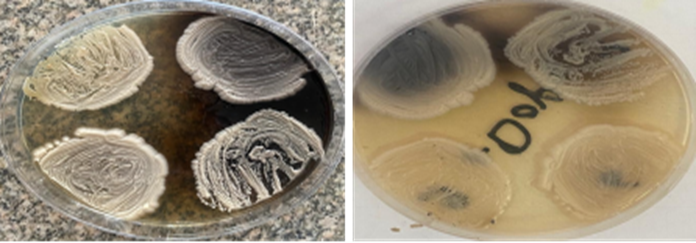 | 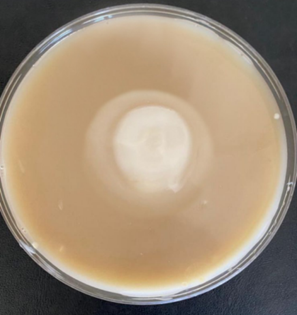 |
| 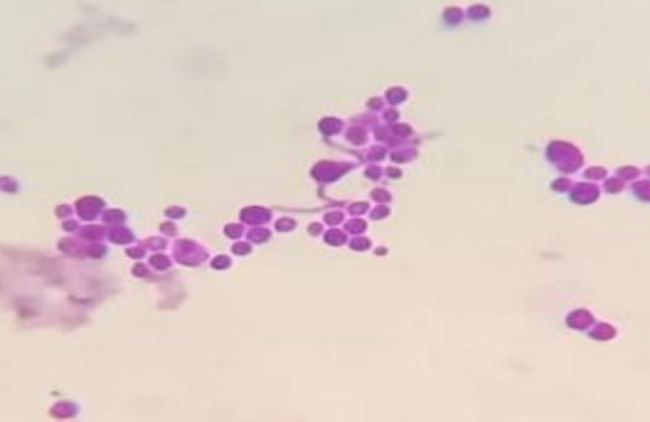 | |

**Figure (supplementary): (1): A-**biofilm scoring by tube method, Lt:1:+++,2:++,3:+,4:-,Rt: positive and negative control, **B**-*Candida* isolates with hemolytic activity, **C-**Melanin production by different *Candida* spp. on MM media supplemented with L-DOPA, **D-**Proteinase production by *Candida* isolates on skimmed milk agar plate, **E-**positive Germ tube test by *candida albicans*.

**Table 1(supplementary):** Effect of antifungal drugs on different isolated *Candida* spp. from different specimens. OR/ Influence of Antifungal drugs on Isolated *Candida* Species Across Various Specimen Types.

| ***Candida* spp** | **Flucytosine** | | **Fluconazole** | | **Caspofungin** | | **Amphotericin B** | | **Micafungin** | | **Voriconazole** | |
| --- | --- | --- | --- | --- | --- | --- | --- | --- | --- | --- | --- | --- |
|  | **No** | **%** | **No** | **%** | **No** | **%** | **No** | **%** | **No** | **%** | **No** | **%** |
| ***C.albicans* (n=48)**   - R - I - S | 0  1  47 | 0.0  2.1  97.9 | 0  2  46 | 0.0  4.2  95.8 | 4  0  44 | 8.3  0.0  91.7 | 0  0  48 | 0.0  0.0  100.0 | 4  0  44 | 8.3  0.0  91.7 | 0  0  48 | 0.0  0.0  100.0 |
| ***C.glabrata* (n=16)**   - R - I - S | 0  0  16 | 0.0  0.0  100.0 | -  -  - | -  -  - | 5  5  6 | 31.25  31.25  37.5 | 0  0  16 | 0.0  0.0  100.0 | 0  0  16 | 0.0  0.0  100.0 | 0  0  16 | 0.0  0.0  100.0 |
| ***C.tropicalis* (n=14)**   - R - I - S | 8  0  6 | 57.1  0.0  42.9 | 0  0  14 | 0.0  0.0  100.0 | 0  0  14 | 0.0  0.0  100.0 | 0  0  14 | 0.0  0.0  100.0 | 2  0  12 | 14.3  0.0  85.7 | 0  0  14 | 0.0  0.0  100.0 |
| ***C.parapsilosis* (n=10)**   - R - I - S | 0  0  10 | 0.0  0.0  100.0 | 4  0  6 | 40.0  0.0  60.0 | 0  0  10 | 0.0  0.0  100.0 | 0  0  10 | 0.0  0.0  100.0 | 0  0  10 | 0.0  0.0  100.0 | 0  0  10 | 0.0  0.0  100.0 |
| ***C.dubliniensis* (n=4)**   - R - I - S | 0  1  3 | 0.0  25.0  75.0 | 0  1  3 | 0.0  25.0  75.0 | 0  0  4 | 0.0  0.0  100.0 | 0  0  4 | 0.0  0.0  100.0 | 0  0  4 | 0.0  0.0  100.0 | 0  0  4 | 0.0  0.0  100.0 |
| ***C.guilliromondii* (n=4)**   - R - I - S | 0  0  4 | 0.0  0.0  100.0 | 0  0  4 | 0.0  0.0  100.0 | 2  0  2 | 50.0  0.0  50.0 | 0  0  4 | 0.0  0.0  100.0 | 0  2  2 | 0.0  50.0  50.0 | 0  0  4 | 0.0  0.0  100.0 |
| ***C.lusitanaie* (n=2)**   - R - I - S | 0  0  2 | 0.0  0.0  100.0 | 0  0  2 | 0.0  0.0  100.0 | 0  0  2 | 0.0  0.0  100.0 | 0  0  2 | 0.0  0.0  100.0 | 0  0  2 | 0.0  0.0  100.0 | 0  0  2 | 0.0  0.0  100.0 |
| ***C.auris* (n=2)**   - R - I - S | 0  0  2 | 0.0  0.0  100.0 | 2  0  0 | 100.0  0.0  0.0 | 2  0  0 | 100.0  0.0  0.0 | 2  0  0 | 100.0  0.0  0.0 | 2  0  0 | 100.0  0.0  0.0 | 2  0  0 | 100.0  0.0  0.0 |

R: Resistant, I: Intermediate, S: Sensitive

**Table 2 (supplementary): Correlation between specimen types and virulence traits in *Candida* spp.**

| **Virulence traits** | | | | | | | | | | | | |
| --- | --- | --- | --- | --- | --- | --- | --- | --- | --- | --- | --- | --- |
| **Type of**  **Specimen** | **Biofilm** | | **Germination** | | **Proteinase** | | **Melanin** | | **Hemolytic**  **activity** | | **Coagulase** | |
|  | **+ve** | **-ve** | **+ve** | **-ve** | **+ve** | **-ve** | **+ve** | **-ve** | **+ve** | **-ve** | **+ve** | **-ve** |
|  | **n**  **(%)** | **n**  **(%)** | **n**  **(%)** | **n**  **(%)** | **n**  **(%)** | **n**  **(%)** | **n**  **(%)** | **n**  **(%)** | **n**  **(%)** | **n**  **(%)** | **n**  **(%)** | **n**  **(%)** |
| **Blood**  **(n=54)** | 18  (56.3) | 36  (52.9) | 34  (60.7) | 20  (45.5) | 34  (65.4) | 20  (41.7) | 20  (58.8) | 34  (51.5) | 26  (59.1) | 28  (50.0) | 24  (57.1) | 30  (51.7) |
| **Urine**  **(n=24)** | 8  (25) | 16  (23.5) | 10  (17.9) | 14  (31.8) | 12  (23.1) | 12  (25.0) | 8  (23.5) | 16  (24.2) | 10  (22.7) | 14  (25.0) | 12  (28.6) | 12  (20.7) |
| **Vaginal swab**  **(n=12)** | 2  (6.3) | 10  (14.7) | 10  (17.9) | 2  (4.5) | 2  (3.8) | 10  (20.8) | 4  (11.8) | 8  (12.1) | 2  (4.5) | 10  (17.9) | 4  (9.5) | 8  (13.8) |
| **Ascitic fluid**  **(n=4)** | 2  (6.3) | 2  (2.9) | 0  (0.0) | 4  (9.1) | 2  (3.8) | 2  (4.2) | 0  (0.0) | 4  (6.1) | 4  (9.1) | 0  (0.0) | 2  (4.8) | 2  (3.4) |
| **Sputum**  **(n=2)** | 0  (0.0) | 2  (2.9) | 0  (0.0) | 2  (4.5) | 0  (0.0) | 2  (4.2) | 0  (0.0) | 2  (3.0) | 0  (0.0) | 2  (3.6) | 0  (0.0) | 2  (3.4) |
| **BAL**  **(n=2)** | 0  (0.0) | 2  (2.9) | 2  (3.6) | 0  (0.0) | 0  (0.0) | 2  (4.2) | 0  (0.0) | 2  (3.0) | 0  (0.0) | 2  (3.6) | 0  (0.0) | 2  (3.4) |
| **Wound swab**  **(n=2)** | 2  (6.3) | 0  (0.0) | 0  (0.0) | 2  (4.5) | 2  (3.8) | 0  (0.0) | 2  (5.9) | 0  (0.0) | 2  (4.5) | 0  (0.0) | 0  (0.0) | 2  (3.4) |
| **Total** | **32**  **(100.0)** | **68**  **(100.0)** | **56**  **(100.0)** | **44**  **(100.0)** | **52**  **(100.0)** | **48**  **(100.0)** | **34**  **(100.0)** | **66**  **(100.0)** | **44**  **(100.0)** | **56**  **(100.0)** | **42**  **(100.0)** | **58**  **(100.0)** |
