# Supplementary figures and images for: Characterization of Candida species isolated from clinical specimens: insights into virulence traits, antifungal resistance and molecular profiles
Source: BMC Microbiol. 2024 Oct 5;24:388. doi: 10.1186/s12866-024-03515-x (PMC11453005; doi:10.1186/s12866-024-03515-x)

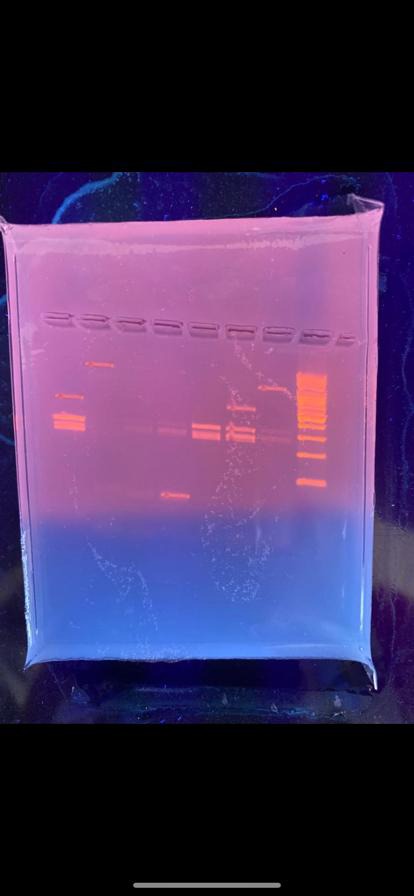

Supplement: Supplementary file 2 — Supplementary Material 2 [file 12866_2024_3515_MOESM2_ESM.jpg]

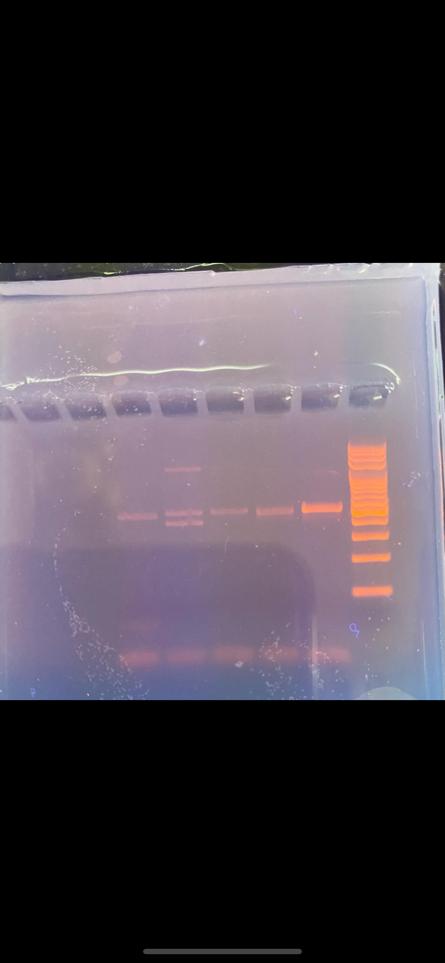

Supplement: Supplementary file 3 — Supplementary Material 3 [file 12866_2024_3515_MOESM3_ESM.jpg]

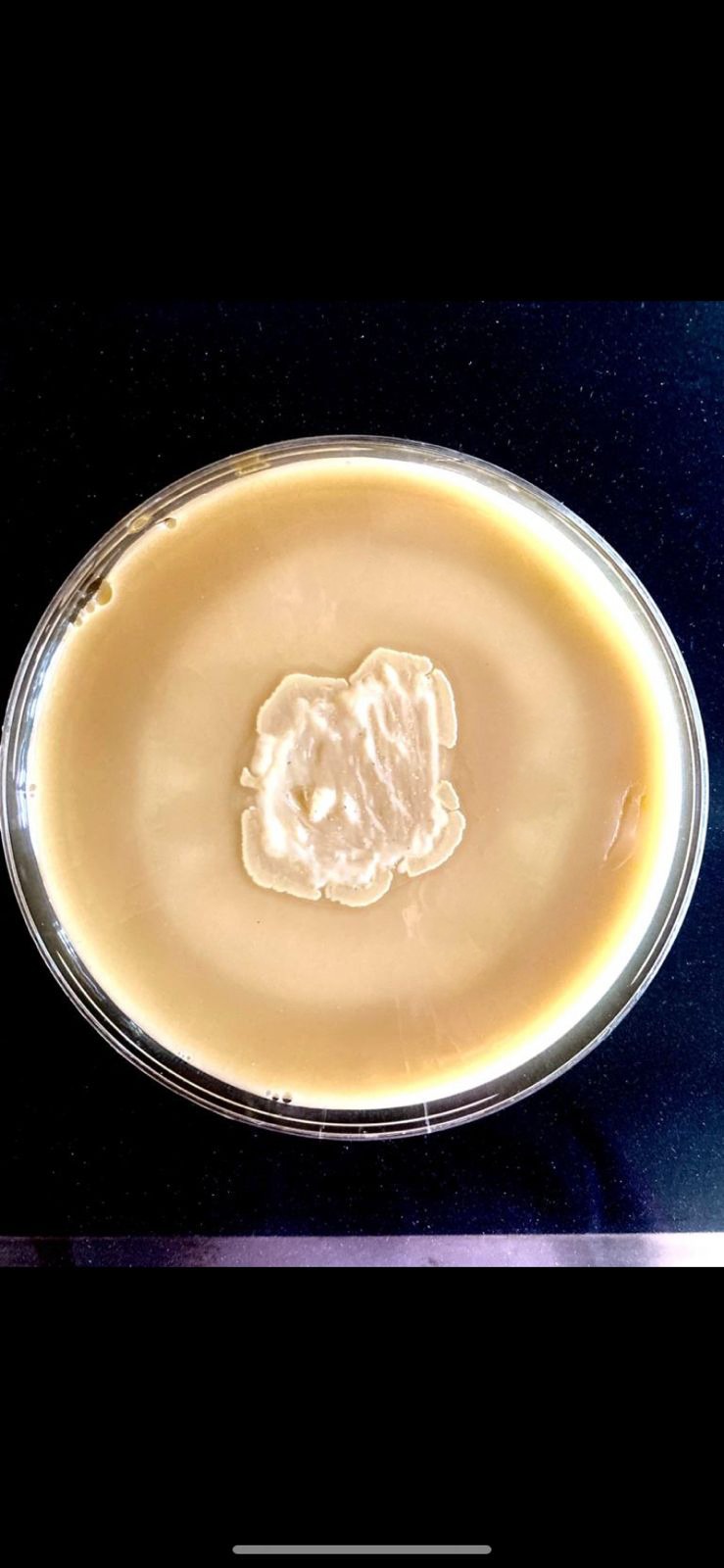

Supplement: Supplementary file 4 — Supplementary Material 4 [file 12866_2024_3515_MOESM4_ESM.jpg]

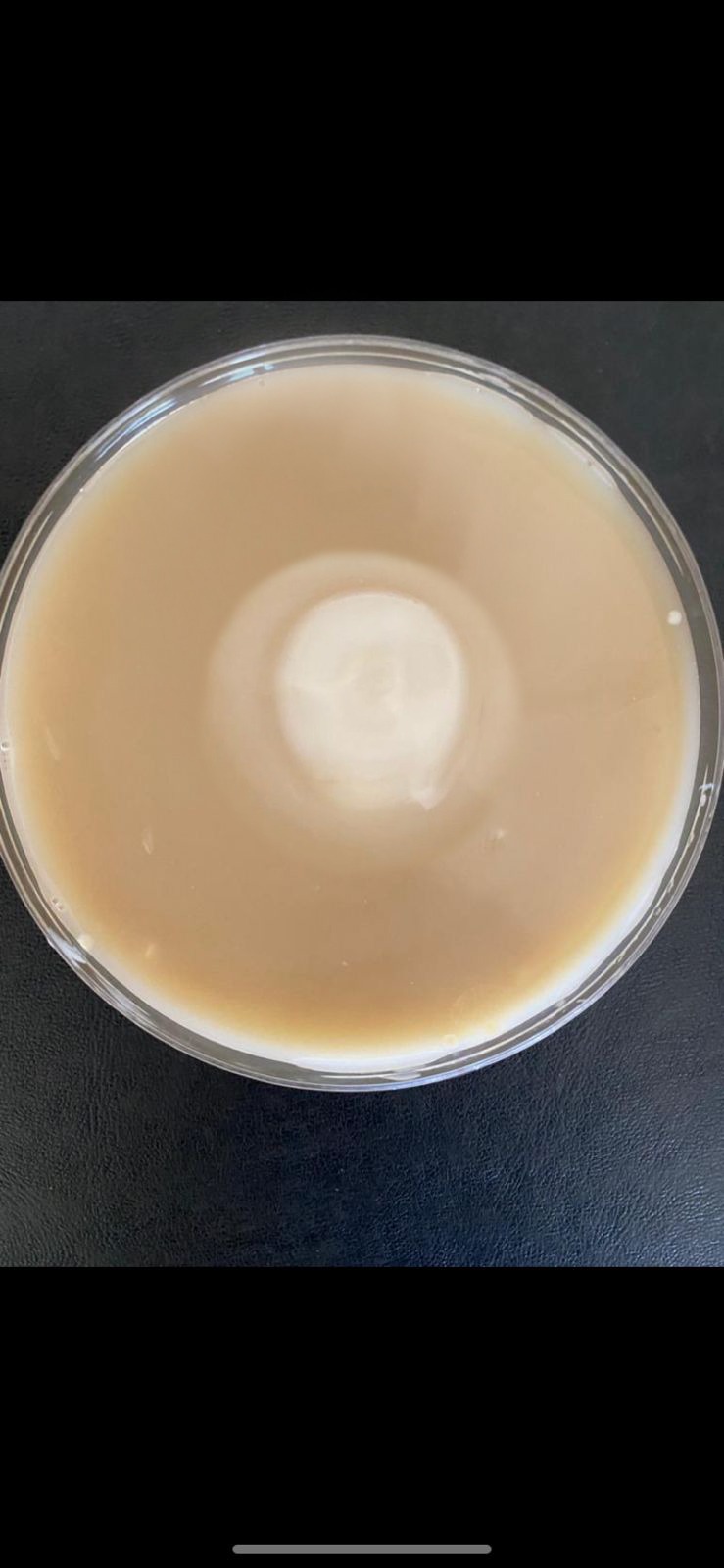

Supplement: Supplementary file 5 — Supplementary Material 5 [file 12866_2024_3515_MOESM5_ESM.jpg]
